# Efficacy of Compounds Isolated from the Essential Oil of *Artemisia lavandulaefolia* in Control of the Cigarette Beetle, *Lasioderma serricorne*

**DOI:** 10.3390/molecules23020343

**Published:** 2018-02-07

**Authors:** Jun Zhou, Kexing Zou, Wenjuan Zhang, Shanshan Guo, Hong Liu, Jiansheng Sun, Jigang Li, Dongye Huang, Yan Wu, Shushan Du, Almaz Borjigidai

**Affiliations:** 1 Technical Center of China Tobacco Guangxi Industrial Co., Ltd., Nanning 530001, Guangxi, China; zhoujuntobacco@126.com (J.Z.); zoukx720611@126.com (K.Z.); liuh6969@sohu.com (H.L.); nnsjs@sina.com (J.S.); ljg110916@sina.com (J.L.); huangdongye8@163.com (D.H.); abelabel@126.com (Y.W.); 2 Beijing Key Laboratory of Traditional Chinese Medicine Protection and Utilization, Faculty of Geographical Science, Beijing Normal University, No. 19 Xinjiekouwai Street, Beijing 100875, China; zwj0729@mail.bnu.edu.cn (W.Z.); guoshanshan@mail.bnu.edu.cn (S.G.); 3 College of Pharmacy, Minzu University of China, No. 27 Zhongguancun South Street, Beijing 100081, China

**Keywords:** natural products, chamazulene, fumigant toxicity, contact toxicity, repellency, tobacco storage

## Abstract

To develop natural product resources to control cigarette beetles (*Lasioderma serricorne*), the essential oil from *Artemisia lavandulaefolia* (Compositae) was investigated. Oil was extracted by hydrodistillation of the above-ground portion of *A. lavandulaefolia* and analyzed using gas chromatography-mass spectrometer (GC-MS). Extracted essential oil and three compounds isolated from the oil were then evaluated in laboratory assays to determine the fumigant, contact, and repellent efficacy against the stored-products’ pest, *L. serricorne*. The bioactive constituents from the oil extracts were identified as chamazulene (40.4%), 1,8-cineole (16.0%), and β-caryophyllene (11.5%). In the insecticidal activity assay, the adults of *L. serricorne* were susceptible to fumigant action of the essential oil and 1,8-cineole, with LC_50_ values of 31.81 and 5.18 mg/L air. The essential oil, 1,8-cineole, chamazulene, and β-caryophyllene exhibited contact toxicity with LD_50_ values of 13.51, 15.58, 15.18 and 35.52 μg/adult, respectively. During the repellency test, the essential oil and chamazulene had repellency approximating the positive control. The results indicated that chamazulene was abundant in *A. lavandulaefolia* essential oil and was toxic to cigarette beetles.

## 1. Introduction

As a cosmopolitan pest of stored-products, the cigarette beetle *Lasioderma serricorne* (Fabricius) (Coleoptera: Ptinidae), occurs widely in the tropical and subtropical regions of the world [1]. It causes serious damage and economic loss to stored animal and plant materials, particularly raw tobacco and tobacco products [2,3]. Adult cigarette beetles chew holes and excavate tunnels in these materials to mate and lay eggs [4]. After the eggs hatch, larvae consume large quantities of food materials, and are responsible for causing significant damage or economic loss [5].

Pest control measures used to protect cured tobacco and tobacco products from infestation by *L. serricorne* commonly rely on the use of pyrethroid and phosphine insecticides, which raise many health and environmental issues [6]. These problems have induced a search for alternative ecologically-safe pest control methods [7]. The application of essential oils or their constituents with low toxicity to non-target organisms is one possible method for effectively preventing insect pests in tobacco warehouses [8]. Numerous essential oils and their constituents isolated from plants (including medicinal herbs, spices, and fruits) have been evaluated successfully for insecticidal or repellent activity against stored product insects, and some are even considered to be more effective than traditionally-used pesticides [9,10,11,12,13,14,15,16,17,18].

Compositae is the most evolved family within the Angiospermae and has the highest diversity of species. Amongst the botanical families recognized by the insecticidal properties of its secondary metabolites, Compositae is one of the most important [19,20]. Within this family, *Artemisia* spp. occur in temperate, cold, and subtropical zones of the northern hemisphere, and is comprised of over 180 species in China [21,22,23]. Many of these species have long been considered to have medicinal value, such as *A. annua*, *A. capillaris*, and *A. argyi*. Furthermore, according to some traditional Chinese medicinal materials, conservation methods, and global modern investigations, some *Artemisia* plants were considered to have effects against various stored-product insects [24,25,26,27,28,29,30,31]. However, few documents examine the toxicity of active compounds from *Artemisia* spp. against *L. serricorne*, despite the known insecticidal properties of this genus [27,28,29,30,31].

*Artemisia lavandulaefolia* DC is a perennial herbaceous plant. Reviewing the literature, the essential oil from the fresh above-ground portion of *A. lavandulaefolia* has been reported to show insecticidal activity against some pests [24,32,33,34,35]. However, only two stored-product insects (maize weevil, *Sitophilus zeamais*, and adzuki bean weevil, *Callosobruchus chinensis*) were examined [24,34,35], and the effectiveness of the various components was not evaluated. In recent years, the reports about the chemical composition of essential oil extracted from *A. lavandulaefolia* in China has increased [24,32,34,35], contributing to speculation of their characteristics of chemical components and finding relations between the activities. Taking the significance of this plant’s resources into consideration, we investigate the repellent and insecticidal activity of the chemical constituents of this essential oil against *L. serricorne*.

## 2. Results

### 2.1. Composition Analysis

Essential oil from *A. lavandulaefolia* was dark blue with a yield of 0.14% (*v*/*w*) and a density of 0.89 g/mL. The results revealed that the oil was mainly constituted of 20 components (accounting for 92.6%). The four main components in the essential oil are chamazulene (40.4%), 1,8-cineole (16.0%), β-caryophyllene (11.5%), and β-farnesene (5.3%). The first three compounds were isolated by silica gel column chromatogram and identified by NMR, which were in accordance with the compounds given by GC-MS (Table 1).

### 2.2. Isolated Compounds 

**Figure 1:**
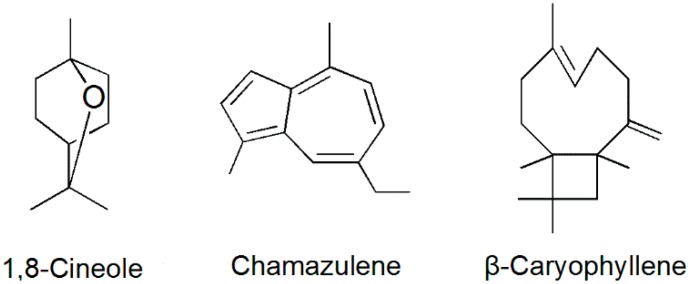
Chemical structures of the three most abundant compounds isolated from the essential oil.

Three compounds were isolated from the *A. lavandulaefolia* for the first time. They were identified as 1,8-cineole, chamazulene, and β-caryophyllene by comparison of their spectral data with those of the literature (^1^H-, ^13^C-NMR, and mass spectra) [36,37,38]. 1,8-Cineole is an oxygenated monoterpenoid. Chamazulene and β-caryophyllene are sesquiterpenes. Their structures are shown in Figure 1.

### 2.3. Bioactivity Analysis

#### 2.3.1. Fumigant and Contact Toxicity

**Figure 2:**
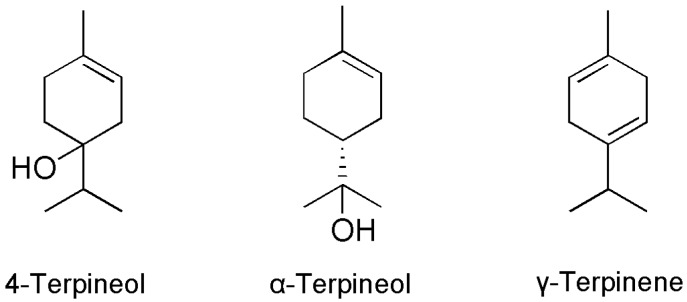
The other three monoterpenoids from the essential oil of *A. lavandulaefolia*.

The essential oil and the main compounds exhibited fumigant and contact toxicity. In addition to the three isolated compounds, some existing data for the other three monoterpenoids (4-terpineol, α-terpineol, and γ-terpinene) that we identified in this essential oil were also listed here to compare their effects to control *L. serricorne* adults. Their structures are shown in Figure 2. Thus, the data of the six compounds and the essential oil, as well as the positive controls, are shown in Table 2.

Although 1,8-cineole showed considerably less fumigant toxicity (LC_50_ = 5.18 mg/L air) than the positive control (phosphine, LC_50_ = 9.23 × 10^−3^ mg/L air), it showed considerable fumigant toxicity against *L. serricorne* adults. The three monoterpenoids all had stronger fumigant activity than the essential oil, particularly α-terpineol had the lowest LC_50_ value against the pest. The four monoterpenoids (4-terpineol, α-terpineol, γ-terpinene and 1,8-cineole) were more effective than the two sesquiterpenoids (chamazulene and β-caryophyllene).

The essential oil of *A. lavandulaefolia* exhibited contact toxicity against *L. serricorne* adults (LD_50_ = 13.51 μg/adult) and it demonstrated less toxicity than the positive control, pyrethrins (LD_50_ = 0.24 µg/adult). Three isolated compounds, chamazulene, 1,8-cineole and β-caryophyllene were also toxic to *L. serricorne* adults, with LD_50_ values of 15.18, 15.58, and 35.52 μg/adult, respectively (Table 2). Moreover, chamazulene possessed almost two times more toxicity than β-caryophyllene.

#### 2.3.2. Repellent Activity

**Figure 3:**
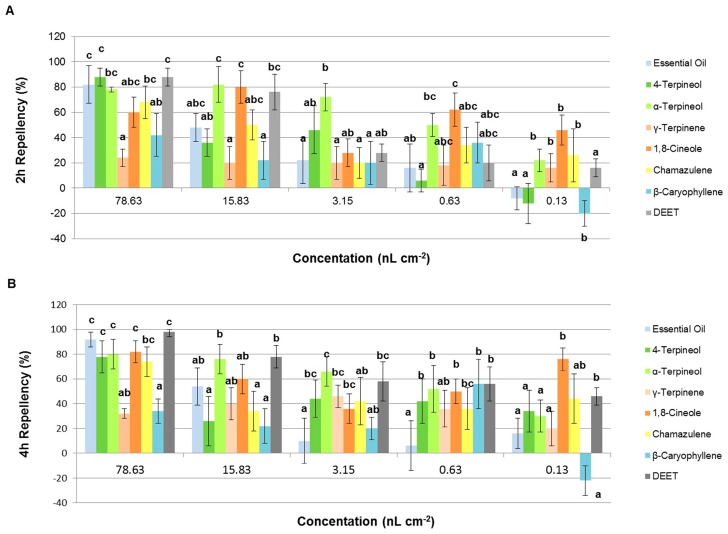
Percentage repellency (PR) of the essential oil and its main constituents against *L. serricorne* after 2 h (**A**) and 4 h (**B**) treatment. Means in the same column followed by the same letters do not differ significantly (*p* > 0.05) in ANOVA and Tukey’s tests. PR was subjected to an arcsine square-root transformation before ANOVA and Tukey’s tests.

*Artemisia lavandulaefolia* essential oil and the six constituents repelled *L. serricorne* adults at the first four concentrations. The results are presented in Figure 3 and Table A1 in Appendix A. Data showed that at doses of 78.63, 15.83, 3.15, and 0.63 nL/cm^2^, the crude essential oil showed the same level of repellency (*p* > 0.05) against *L. serricorne* adults at 2 h after exposure. Moreover, at 4 h after exposure, the essential oil showed the same level of repellency (*p* > 0.05) against *L. serricorne* adults at 78.63 and 15.83 nL/cm^2^. At tested concentration of 78.63 nL/cm^2^, the main ingredient chamazulene repelled *L. serricorne* adults effectively (*PR* > 80%), however, the effect reduced greatly (*PR* < 50%) when the concentration diluted. The repellent activity of β-caryophyllene was relatively weak (*PR* < 60%) at all concentrations. It even exhibited an attracting action at the concentration of 0.13 nL/cm^2^.

As for the four monoterpenoids, the three oxygenated ones (4-terpineol, α-terpineol, and 1,8-cineole) showed the same or better efficiency than γ-terpinene. α-Terpineol possessed a better effect than both 4-terpineol and 1,8-cineole at the doses of 78.63, 15.83, 3.15, and 0.63 nL/cm^2^. At the lowest tested concentration (0.13 nL/cm^2^), 1,8-cineole showed much stronger repellency (76%) than the positive control, DEET (*N*,*N*-diethyl-3-methyl-benzamide, *PR* = 46%), at 4 h after exposure.

## 3. Discussion

### 3.1. Composition of the Essential Oil

The chemical composition of the essential oil of *A. lavandulaefolia* in the present study was different from previous reports. For example, β-caryophyllene (15.5%), β-thujone (13.6%), 1,8-cineole (13.1%), and β-farnesene (12.3%) were the main volatile components of this plant from Beijing, China [24]. Extracted by the same method, 1,8-cineole (10.7%), α-terpineol (5.3%), 4-carene (4.0%), 4-methyl-1-(1-methylethyl)-bicyclo[3.1.0]hexan-3-one (3.6%), caryophyllene oxide (2.6%), and β-caryophyllene (2.2%) were the dominant compounds in the sample from Changchun, China [34]. However, the major compounds in the samples collected in Mt. Unnamsan in Korea were β-caryophyllene (16.1%), *cis*-chrysanthenol (7.0%), 1,8-cineole (5.6%), borneol (5.3%), and β-farnesene (5.1%) [41]. It can be deduced from these results that 1,8-cineole and β-caryophyllene were common constituents in *A. lavandulaefolia* [24,34,41,42]. Nevertheless, chamazulene had not been previously identified as a main component in this plant, which perhaps suggested a new chamazulene chemotype, but more evidence is needed to confirm this idea. In addition, we have not identified *cis*-chrysanthenol in the samples collected in China [24,34]. Some literature analyses showed that *A. absinthium* harvested from June to November contained varying amounts of cis-chrysanthenol, which might suggest that the geographical environment and harvest time are the important factors in this plant [43].

It is important to point out that, our work also found that the species and content of the chemical compounds according to the relation of essential oils in yarrow or milfoil (*Achillea millefolium* L., Asteraceae or Compositae). That is, a strong negative correlation between the amounts of β-pinene and β-caryophyllene, β-pinene, and 1,8-cineole, as well as germacrene D and β-caryophyllene. Additionally, the strong negative correlation between the amounts of chamazulene and (*E*)-nerolidol, and a moderate negative correlation between the amounts of chamazulene and sabinene were also verified partly in this paper, because there were neither (*E*)-nerolidol nor sabinene found in this essential oil [44]. Further research is needed to obtain more samples and to find more evidence.

### 3.2. Structure-Bioactivity Relationship of the Four Monoterpenoids

Three compounds we isolated in this work are common components in essential oils. In our previous research, the essential oil of *Dendranthema indicum* were extracted, and then the same three isolated compounds (1,8-cineole, chamazulene, and β-caryophyllene) were also isolated [45]. In this essential oil, monoterpenoids accounting for over 20%, and the proportion of the other three listed monocyclic ones (4-terpineol, α-terpineol, and γ-terpinene) were nearly 7%.

By comparing the structures with their bio-effects, the structure-bioactivity relationships were apparent. The four monoterpenoids shared the same basic parent structure (monoterpenoid skeleton) but with different substitution patterns. As for the fumigant activity, the molecules with oxygen bridges between the ring and the substituent group (1,8-cineole) had less fumigant toxicity than the one branched chain oxygenous groups (α-terpineol), but more effective than the compounds that had oxygenous groups linked to the saturated carbon ring directly (4-terpineol). However, for 1,8-cineole, the compounds with oxygen bridges had the weakest contact toxicity than the other monoterpenoids. 4-Terpineol had oxygenous groups linked to the saturated carbon ring directly, which possessed the highest contact toxity. For the repellent activities against *L. serricorne* adults, the monoterpenoid without the substitutional group (γ-terpinene) had the relatively weakest effects. Overall, the activities of the monoterpenoids might depend on the number and position of the oxygenous groups. This result may have implications for the chemical structural modification of leading compounds.

### 3.3. Characteristics of Chamazulene

Among the three isolated compounds, chamazulene is a quite special one. Converted by nonvolatile sesquiterpene lactone matricin, this compound is formed during distillation. It is a bicyclic C14 aromatic hydrocarbon with system of conjugated double bonds, which the essential oil appears as a deep blue color [46].

Chamazulene has a variety of pharmacological activities, such as an anti-inflammatory and antioxidant [46,47]. Its insecticide activity was also proven in our earlier study, which had contact activity against *Stegobium paniceum* and *Tribolium castaneum* (LD_50_ = 4.30 and 29.52 µg/adult), respectively [45]. Its fumigant, contact, and repellent effects against the cigarette beetle are found for the first time. Furthermore, chamazulene is a characteristic composition from German chamomile and some chemotypes of yarrow [46]. However, there were few records about this compound in *Artemisia* in China. Here, the chamazulene accounts for over 40% of the essential oil, which suggests *A. lavandulaefolia* is a valuable plant resource.

As early as 2010, Dr. Yuan had already analyzed the bioactivity, mechanism, and security of the essential oil from *A. lavandulaefolia* systematically, and demonstrated the prospects of this plant’s resource development [48]. This work finds the essential oil of the above-ground portion of *A. lavandulaefolia* and its main compounds had toxic effects to *L. serricorne* adults. Further studies will continue to explore more chemical constituents in this plant.

## 4. Materials and Methods

### 4.1. Material

#### 4.1.1. Chemicals

Silica gel (160–200 or 200–300 mesh, Qingdao Marine Chemical Plant, Qingdao, China), were used for column chromatography (CC), and silica gel G (Qingdao Marine Chemical Plant, China) was used for TLC. DEET (positive control in repellent test) and C_5_–C_36_ *n*-alkanes were purchased from Innochem, China. Fluon (Beijing Sino-Rich Co., Beijing, China) was used as an escape-proof coating.

#### 4.1.2. Plants

*Artemisia lavandulaefolia* were grown in Jining City (35.23° N latitude and 116.33° E longitude), Shandong Province, China. Its above-ground portion (10.0 kg) of collected in October 2013. The sample were air-dried for one week.

#### 4.1.3. Insects

*Lasioderma serricorne* were reared in jars containing diets (wheat feed/yeast, 10:1, *w*/*w*) in incubators in the dark (29 ± 1 °C, 70–80% r.h.). All the insects used in all the experiments were about 5 ± 2 days old regardless of gender.

### 4.3. Extraction and Analysis of the Essential Oil 

The oil was hydrodistilled for 6 h using a modified Clevenger-type apparatus and then stored in an airtight container in a refrigerator at 4 °C. Analysis of the essential oil by Gas Chromatography-Mass Spectrometer (GC-MS) and Gas Chromatography-Flame Ionization Detector (GC-FID) was conducted on a Thermo Finnigan Trace DSQ instrument (Waltham, MA, USA) equipped with a flame ionization detector and an HP-5MS capillary column (30 m × 0.25 mm × 0.25 μm). The carrier gas was helium and the flow rate was 1.0 mL/min. The injector temperature was maintained at 250 °C. The essential oils were diluted to 1% with n-hexane and 1 µL was injected into the gas chromatograph. Spectra were scanned from 50 to 550 *m*/*z*. Most constituents was identified by comparison of their mass spectra with those stored in libraries (NIST 05, Standard Reference Data, Gaithersburg, MD, USA and Wiley 275, Wiley, New York, NY, USA) [49]. Further identification were identified by comparison of their retention indices with those reported in the literature. The retention indices were determined in relation to a homologous series of *n*-alkanes (C_5_–C_36_) under the same operating conditions. Relative percentages of each oil component were determined by GC-FID via the percentage peak area calculations.

### 4.4. Purification of Three Compounds

The essential oil (10 mL) was chromatographed on a silica gel column (50 × 500 mm) by eluting with petroleum ether first, then with petroleum ether-ethyl acetate, and last with ethyl acetate. Fractions (200 mL) were collected, and fractions’ profiles were combined to yield 38 fractions on the basis of Thin Layer Chromatography (TLC) analysis. Three fractions (3–8, 10–15, 20–26) were further purified until the pure compounds for determining structure as chamazulene (2.42 g), 1,8-cineole (0.95 g), and β-caryophyllene (0.63 g) were obtained. The isolated compounds were elucidated based on NMR. ^1^H- and ^13^C-NMR spectra were recorded on Bruker Avance DRX 500 instruments using CDCl_3_ as the solvent, with tetramethylsilane (TMS) as the internal standard.

### 4.5. Bioactivity Assay

#### 4.5.1. Fumigant Toxicity

The fumigant activity assay references the testing method of Liu and Ho [50]. The essential oil and chamazulene were tested in this work. The data of other compounds were not. They were obtained from other papers by our research group. A Whatman filter paper (diameter 2.0 cm) was put inside of the screw cap of a glass vial (diameter 2.5 cm, height 5.5 cm, volume 25 mL). Each paper was impregnated with a 10 μL dilution (serial diluted to five concentrations by *n*-hexane). Then the cap was placed tightly on the glass vial (contained 10 insects). *n*-Hexane was used as a control. Five replicates were carried out for all treatments and controls. The insects were considered dead if their appendages did not move when probed with a camel brush after 24 h. The LC_50_ values were calculated using Probit analysis [51].

#### 4.5.2. Contact Toxicity

The contact toxicity was also measured as topical application method proposed by Liu and Ho [50]. Serial dilutions of the essential oil and chamazulene (five concentrations) were prepared in *n*-hexane. Dilutions (aliquots of 0.5 μL) were applied topically to the dorsal thorax of the insects. Five replicates were carried out for all treatments and controls (using *n*-hexane). Fifty adults in replicates with ten insects per replication were treated for the control and for each concentration of the samples. Mortality was recorded after 24 h and the LD_50_ values were calculated using Probit analysis [51]. The contact toxicity of other compounds were quoted from other works by our research team.

#### 4.5.3. Repellency Tests

The area preference method were using to test the repellent activity [52]. Five solutions (78.63, 15.83, 3.15, 0.63, and 0.13 nL/cm^2^) were prepared by diluting the essential oil and the six comstituents with *n*-hexane. Each solution was applied to half a filter-paper disc as uniformly as possible with a micropipette. Petri dishes (9 cm in diameter) were used to confine cigarette beetles during the experiment. Filter paper (9 cm in diameter) was cut in half. The other half (control) was treated with 500 μL *n*-hexane. Both the two halves were then air-dried to evaporate the solvent completely. The tested and control halves were attached with solid glue to carefully remake a full disk . Twenty adult beetles of mixed sex were released separately at the center of each filter paper disc. The dishes were then covered and transferred to an incubator at room temperature. Ten replications were used for each concentration. After twenty insects were released on the center of each filter paper disk, the dishes were then covered and transferred to the incubator. The experiment was repeated three times. Observations on the number of insects present on both the treated (Nt) and untreated (Nc) halves were recorded after 2 and 4 h. The percent repellency (PR) was then calculated using the formula:

PR (%) = [(Nc − Nt)/(Nc + Nt)] × 100

The percentage was subjected to an arcsine square-root transformation. By using SPSS 20.0 (IBM, New York, NY, USA), analysis of variance (One-Way ANOVA) and Tukey’s test were conducted.

## 5. Conclusions

In this paper, we reported three isolated compounds from the essential oil of the above-ground portion of *A. lavandulaefolia* and their activities against cigarette beetles for the first time. Our work finds chamazulene was abundant in *A. lavandulaefolia* essential oil and exhibited contact and repellent effects. As valuable resources, *A. lavandulaefolia* and chamazulene deserves further development.

## Figures and Tables

**Table 1:** Results of chemical composition of the essential oil.

Peak No.	Components	RI ^a^	RA ^b^ (%)	Identification Methods ^c^
**1**	*p*-Xylene	860	0.6	MS, RI
**2**	Santolina triene	900	1.9	MS, RI
**3**	β-Pinene	974	1.5	MS, RI
**4**	*o*-Cymene	1014	0.1	MS, RI
**5**	1,8-Cineole	1031	16.0	MS, RI, NMR
**6**	γ-Terpinene	1056	0.6	MS, RI, Co
**7**	Sabinenehydrate	1071	1.1	MS, RI
**8**	Isoterpinolene	1085	0.1	MS, RI
**9**	Camphor	1143	0.3	MS, RI
**10**	4-Terpineol	1175	4.0	MS, RI, Co
**11**	α-Terpineol	1188	2.0	MS, RI, Co
**12**	γ-Elemene	1397	0.8	MS, RI
**13**	β-Caryophyllene	1451	11.5	MS, RI, NMR
**14**	α-Caryophyllene	1454	0.9	MS, RI, Co
**15**	β-Farnesene	1457	5.3	MS, RI
**16**	Germacrene D	1479	2.7	MS, RI
**17**	Valencene	1504	1.3	MS, RI
**18**	Spathulenol	1523	1.1	MS, RI
**19**	Caryophylladienol II	1644	0.4	MS, RI
**20**	Chamazulene	1735	40.4	MS, RI, NMR
	Total		92.6	

^a^ Retention index (RI) relative to the homologous series of n-hydrocarbons on the HP-5 MS capillary column; ^b^ Relative area (peak area relative to the total peak area); ^c^ MS = mass spectrum. Co = co-injection with standard compound. NMR = nuclear magnetic resonance.

**Table 2:** Results of insecticidal effects of essential oil and its main components against *L. serricorne* adults.

Toxicities	Treatments	Concentrations (%)	LC_50_^e^ (μg/mL Air); LD_50_^f^ (μg/adult)	95% FL ^g^ (μg/mL Air); (μg/Adult)	χ^2^	*p*-Value
**Fumigant**	*A. lavandulaefolia*	3.95–20.00	31.81	28.17–35.92	6.46	0.985
	4-Terpineol ^a^	1.98–10.00	6.90	6.04–7.84	24.84	0.963
	α-Terpineol ^a^	1.98–10.00	3.27	3.17–3.38	8.39	0.998
	γ-Terpinene ^b^	1.98–10.00	11.93	10.54–13.51	8.34	0.998
	1,8-Cineole ^c^	0.99–5.00	5.18	4.63–5.70	16.79	0.951
	Chamazulene	0–50.00	–	–	–	–
	β-Caryophyllene ^c^	0–50.00	–	–	–	–
	Phosphine ^d^	7.20 × 10^−3^–11.12 × 10^−3^	9.23 × 10^−3^	7.13 × 10^−3^–11.37 × 10^−3^	11.96	0.971
**Contact**	*A. lavandulaefolia*	1.98–10.00	13.51	11.51–15.40	5.78	0.982
	4-Terpineol ^a^	0.99–5.00	8.62	7.38–9.85	12.65	0.976
	α-Terpineol ^a^	0.59–3.00	11.99	10.42–13.42	18.96	0.703
	γ-Terpinene ^b^	1.98–10.00	14.13	11.82–16.39	13.36	0.944
	1,8-Cineole ^c^	1.98–10.00	15.58	12.88–18.02	15.18	0.935
	Chamazulene	1.98–10.00	15.18	13.31–17.02	6.39	0.961
	β-Caryophyllene ^c^	3.95–20.00	35.52	31.89–39.54	15.41	0.927
	Pyrethrins ^d^	0.01–0.40	0.24	0.16–0.35	17.36	0.791

^a^ Data from You et al. [27]; ^b^ Data from Wang et al. [39]; ^c^ Data from Zhang et al. [31]; ^d^ Data from Yang et al. [40]; ^e^ 50% of Lethal Concentration; ^f^ 50% of Lethal Dose; ^g^ Fiducial Limits.

## Appendix A

**Table A1:** Percentage repellency (PR) after two exposure times (2 h and 4 h) for the essential oil and its constituents against *L. serricorne* adults ^a^.

Times	Treatments	Concentrations (nL/cm^2^)				
		78.63	15.83	3.15	0.63	0.13
2 h	*A. lavandulaefolia*	82 ± 15 c	48 ± 11 abc	22 ± 18 a	16 ± 19 ab	−8 ± 9 a
	4-Terpineol	88± 7 c	36 ± 11ab	46 ± 19 ab	6 ± 9 a	−12 ± 16 a
	α-Terpineol	78 ± 2 bc	82 ± 14 c	72 ± 11 b	50 ± 9 bc	22 ± 9 b
	γ-Terpinene	24 ± 7 a	20 ± 13 a	20 ± 13 a	18 ± 16 abc	16 ± 11 b
	1,8-Cineole	60 ± 12 abc	80 ± 13 c	28 ± 11 ab	62 ± 13 c	46 ± 12 b
	Chamazulene	68 ± 13 bc	50 ± 12 abc	20 ± 12 a	34 ± 14 abc	26 ± 21 b
	β-Caryophyllene	42 ± 17 ab	22 ± 15 a	20 ± 17 a	36 ± 16 abc	−20 ± 10 b
	DEET	88 ± 7 c	76 ± 14 bc	28 ± 7 ab	20 ± 14 abc	16 ± 7 b
4 h	*A. lavandulaefolia*	92 ± 6 c	54 ± 15 ab	10 ± 18 a	6 ± 20 a	16 ± 12 a
	4-Terpineol	78 ± 13 c	26 ± 20 a	44 ± 15 bc	42 ± 18 b	34 ± 17 a
	α-Terpineol	80 ± 12 c	76 ± 12 b	66 ± 12 c	52 ± 19 b	30 ± 13 a
	γ-Terpinene	32 ± 4 ab	40 ± 13 ab	46 ± 9 bc	36 ± 15 ab	20 ± 14 a
	1,8-Cineole	82 ± 9 c	60 ± 12 ab	36 ± 12 bc	50 ± 10 b	76 ± 9b
	Chamazulene	74 ± 12 bc	34 ± 16 a	42 ± 19 ab	36 ± 17 bc	44 ± 20 ab
	β-Caryophyllene	34 ± 10 a	22 ± 14 a	20 ± 9 ab	56 ± 20 b	−22 ± 12 a
	DEET	98 ± 4 c	78 ± 9 b	58 ± 16 bc	56 ± 14 b	46 ± 7 ab

^a^ Means in the same column (2 h and 4 h, respectively) followed by the same letters do not differ significantly (*p* > 0.05) in ANOVA and Tukey’s tests. PR was subjected to an arcsine square-root transformation before ANOVA and Tukey’s tests.
